# How to improve the safety of bicortical pedicle screw insertion in the thoracolumbar vertebrae: analysis base on three-dimensional CT reconstruction of patients in the prone position

**DOI:** 10.1186/s12891-020-03473-1

**Published:** 2020-07-07

**Authors:** Chao Xu, Qingxian Hou, Yanchen CHU, Xiuling Huang, Wenjiu Yang, Jinglong Ma, Zhijie Wang

**Affiliations:** 1 Qingdao University Medical College, Qingdao, 266071 Shandong China; 2 Department of Spine Surgery, The Affiliated Hospital of Qingdao University, Qingdao, 266555 Shandong China; 3 Clinical Labororatory, The Affiliated Hospital of Qingdao University, Qingdao, China

**Keywords:** Pedicle screw insertion, Bicortical anchorage, Thoracic and lumbar spine disease, Aorta, Inferior vena cava, Prone CT three-dimensional reconstruction

## Abstract

**Background:**

Through the comparison of three-dimensional CT reconstruction between the supine position and the prone position, the relative position of thoracolumbar great vessels and vertebral body was studied, and the shortest safe distance between them was measured to improve the safety of bicortical pedicle screw insertion and reduce the risk of vascular injury.

**Methods:**

Forty adults were selected to participate the research. Three-dimensional reconstruction of thoracolumbar (T9-L3) CT was performed in the prone position and the supine position. The relative distance between the Aorta/Inferior Vena Cava (IVC) and vertebral body was obtained as AVD/VVD respectively. The relative angle of the Aorta/ IVC and the vertebral body was calculated as ∠AOY/∠VOY. Self-controlled experiments were carried out in the prone and the supine positions, and the data obtained were analyzed using SPSS 22.0 statistical software.

**Results:**

The AVD of the prone position and the supine position was the shortest at T12 (3.18 ± 0.68 mm), but the difference was not statistically significant. The aorta of the T9-L3 segment was shifted from the anterolateral to the anteromedial. The ∠AOY of the other groups differed significantly between the prone and supine positions in all vertebrae except T12 and L1 (*P* < 0.05), and the aorta in the prone position was more anteromedial than that of supine position.

With regard to VVD/∠VOY, there was no significant difference between the prone and supine positions (*P* ≥ 0.05), and the minimum VVD of L3 segment is greater than 5.4 mm. The IVC has no obvious mobility and is fixed in the range of 20 ° ~ 30 ° near the midline.

**Conclusion:**

When using bicortical anchoring of pedicle screws, it is safe to ensure that the protruding tips of the screw is less than 3 mm. Due to the mobility of the aorta in different postures and individual differences in anatomy, the prone position CT can help doctors to make better preoperative plans and decisions.

## Background

Pedicle screw fixation is currently the first choice for the treatment of thoracolumbar spine diseases [1]. In the process, most clinicians believe that it is appropriate to insert the screw into 80% of the depth of the bone-screw channel [2, 3].

However, in treating elderly patients, the pedicle screw bicortical fixation is commonly used. The deeper the screw is placed, the larger the contact area between the screw and the bone is, and the stress is dispersed throughout the hard anterior cortex, thus strengthening the screw fixation. The enhanced internal fixation will reduce the screw loosening, displacement and pullout, and improves the success rate of the operation [4–9].

However, this technique is at risk of damaging the blood vessels in the anterior part of the vertebral body [10–12]. To evaluate this risk and safely implement bicortical screw, the present study used imaging to examine the anatomical relationship between the great vessels and the vertebral body, and it also explored ways to improve the accuracy of bicortical fixation and reduce the risk of vascular injury.

## Methods

### Participants

Forty adults were selected to participate in the study: twenty men and twenty women between ages of 21 and 76 with a mean age of 53.4 years old. All participants had no thoracolumbar deformities, major vascular malformations, and anterior thoracolumbar vascular lesions, and none of them had any history of retroperitoneal surgery or thoracolumbar surgery.

### Materials

Computed tomography was carried out by using PHILIPS brilliance iCT 256-row spiral CT, with PHILIPS image post-processing system. All subjects were injected with iodine contrast agent, and statistics were analyzed by using SPSS 22.0 statistical software.

### Methods

The thoracolumbar region was reconstructed in three dimensions CT, with the patients in the prone position and the supine position respectively. In order to obtain a clearer image, iodine contrast agent was injected during the scanning process for angiography. The collected images were reconstructed by Philips ICT image post-processing workstation.

**Fig. 1:**
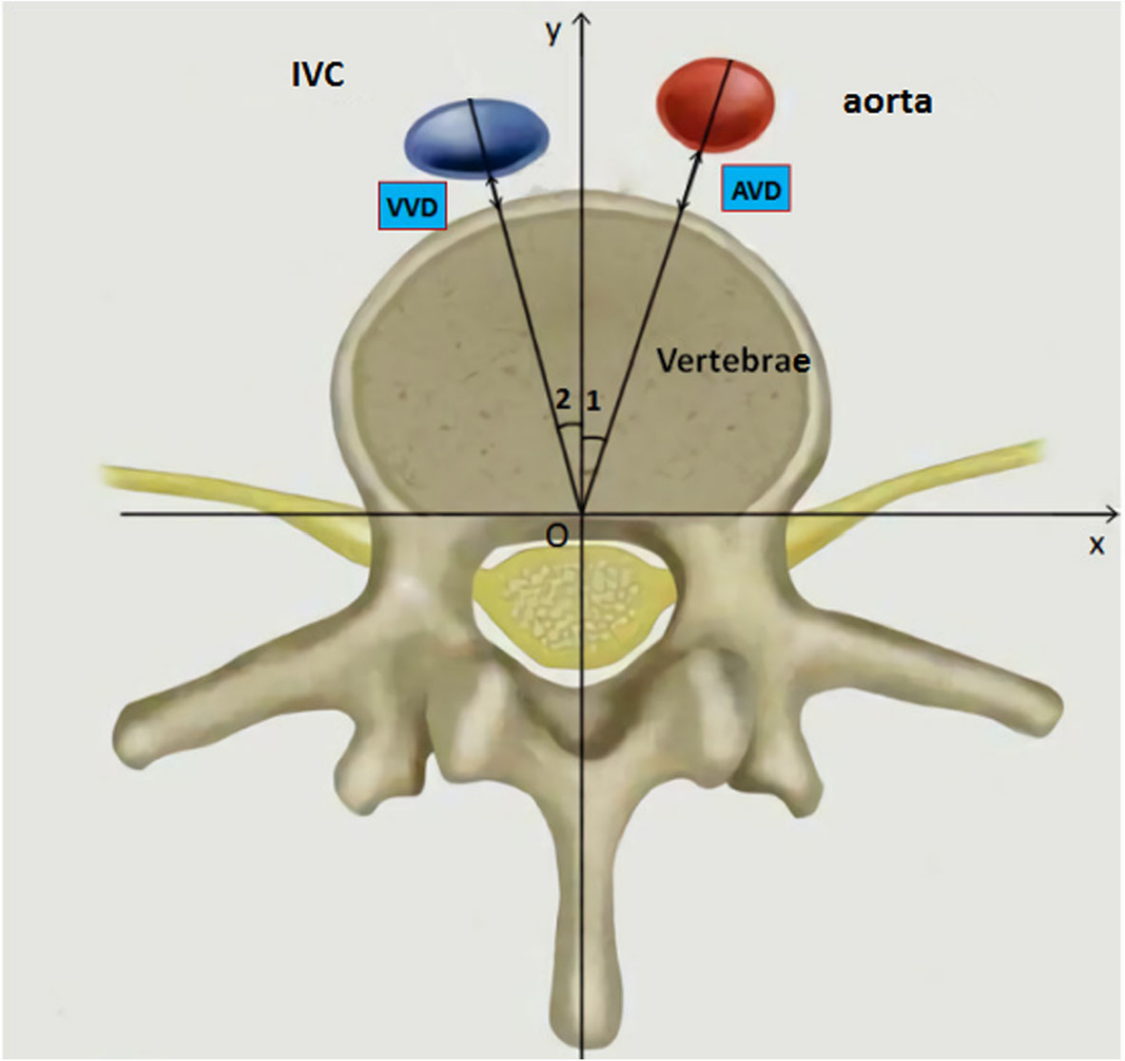
Schematic showing the measured parameters: AVD = the relative distance between the Aorta and vertebral body: VVD = the relative distance between the Inferior Vena Cava and vertebral body; ∠1 = ∠AOY: The relative angle between aorta and vertebral body; ∠2 = ∠VOY: The relative angle between IVC and vertebral body

**Fig. 2:**
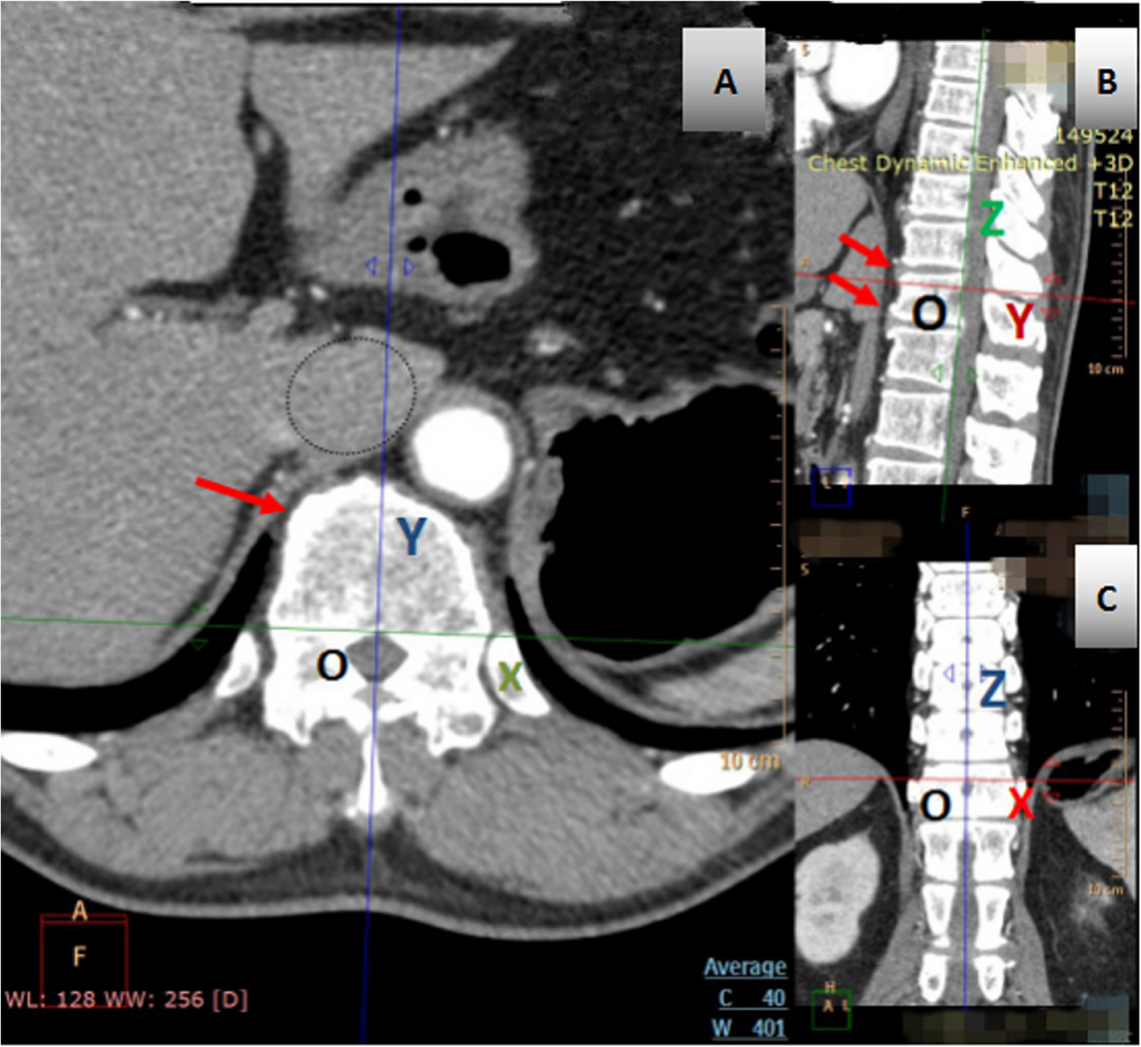
Schematic showing image localization method for T12 level in the supine position. **a**, **b** shows that labial hyperosteogeny may occur on the upper and lower margin of the vertebral body. A shows that the boundary between the IVC and liver tissue passing through the hepatic vena cava sulcus is not obvious

Fig.1 showed the axial image of the optimal pedicle screw trajectory plane. The following measurements were acquired: the angle of great vessels relative to the vertebral body (∠AOY/∠VOY; Fig. 1), and the shortest distance of between the great vessels and vertebral body in this direction. (AVD/VVD),

The experiment was designed to obtain an axial image of the optimal pedicle screw trajectory plane, which was perpendicular to the posterior plane of the vertebral body. In order to facilitate observation and measurement, the reference line was marked as axis X, axis Y, axis Z, and origin O. The X and Z axes passed through the posterior plane of the vertebral body, X axis passed through the center of the pedicle, and the Y axis passed through the midline of the vertebral body. (Fig.1, Fig.2).

**Fig. 3:**
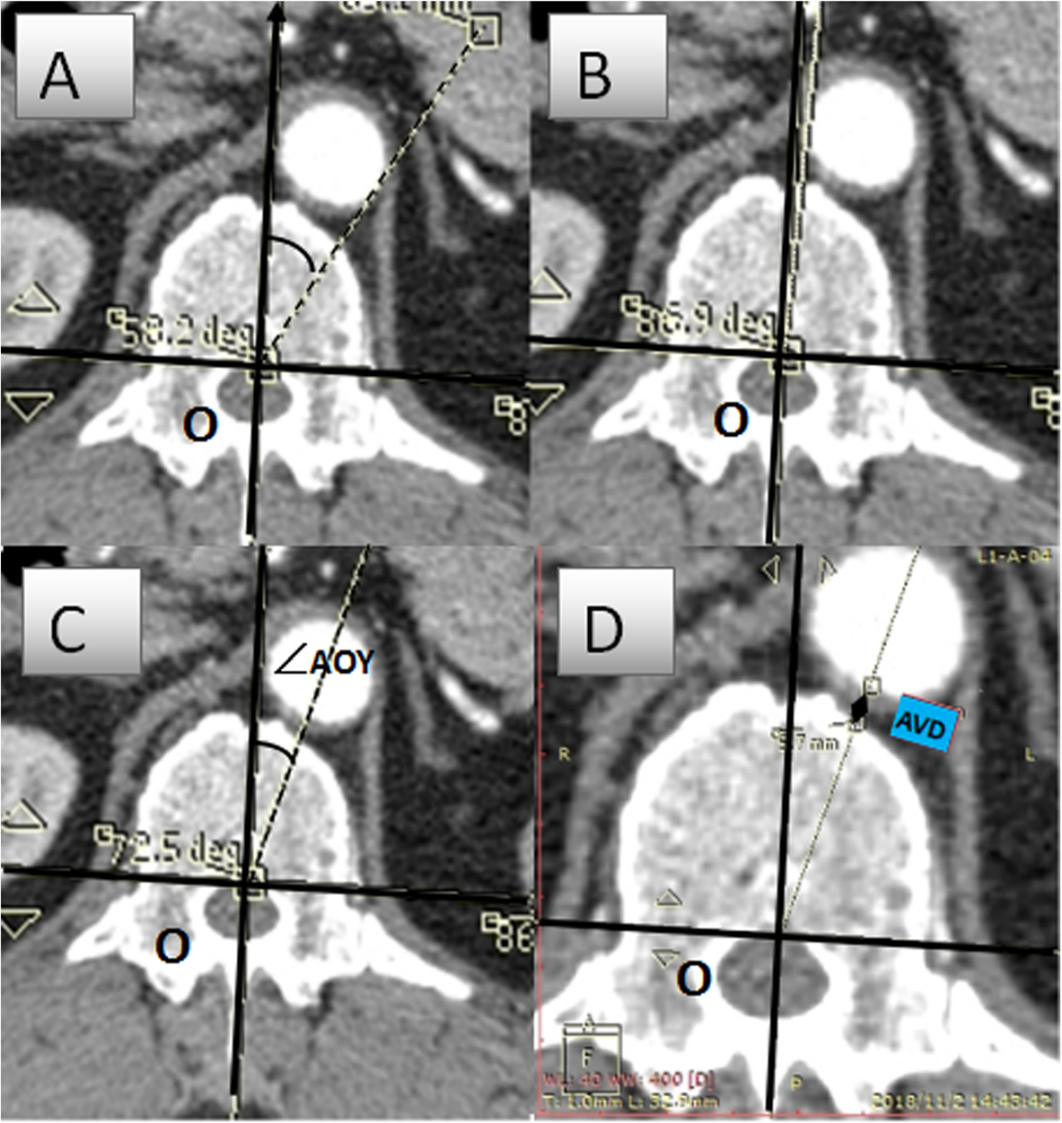
Schematic diagram of data measurement method

On the axial plane, the Y axis was marked as 0°. The ∠AOY/∠VOY of the great vessel refers to the average angle between the tangent point on both sides of the vessel and the origin O relative to the Y axis. The AVD/VVD was measured in the direction of the angle of ∠AOY/∠VOY. (Fig.3).

Self-controlled experiments were carried out on the changes of posture in the prone and the supine positions. The data obtained were analyzed by using SPSS 22.0 statistical software. When the *P* value was less than 0.05, the difference was defined as statistically significant. The experimental data were shown by the table of mean ± standard deviation. Intra-observer reliability was assessed by calculating the spearman correlation coefficient for repeated measurements.

## Results

### The relative position of aorta and vertebral body (AVD/∠AOY)

**Fig. 4:**
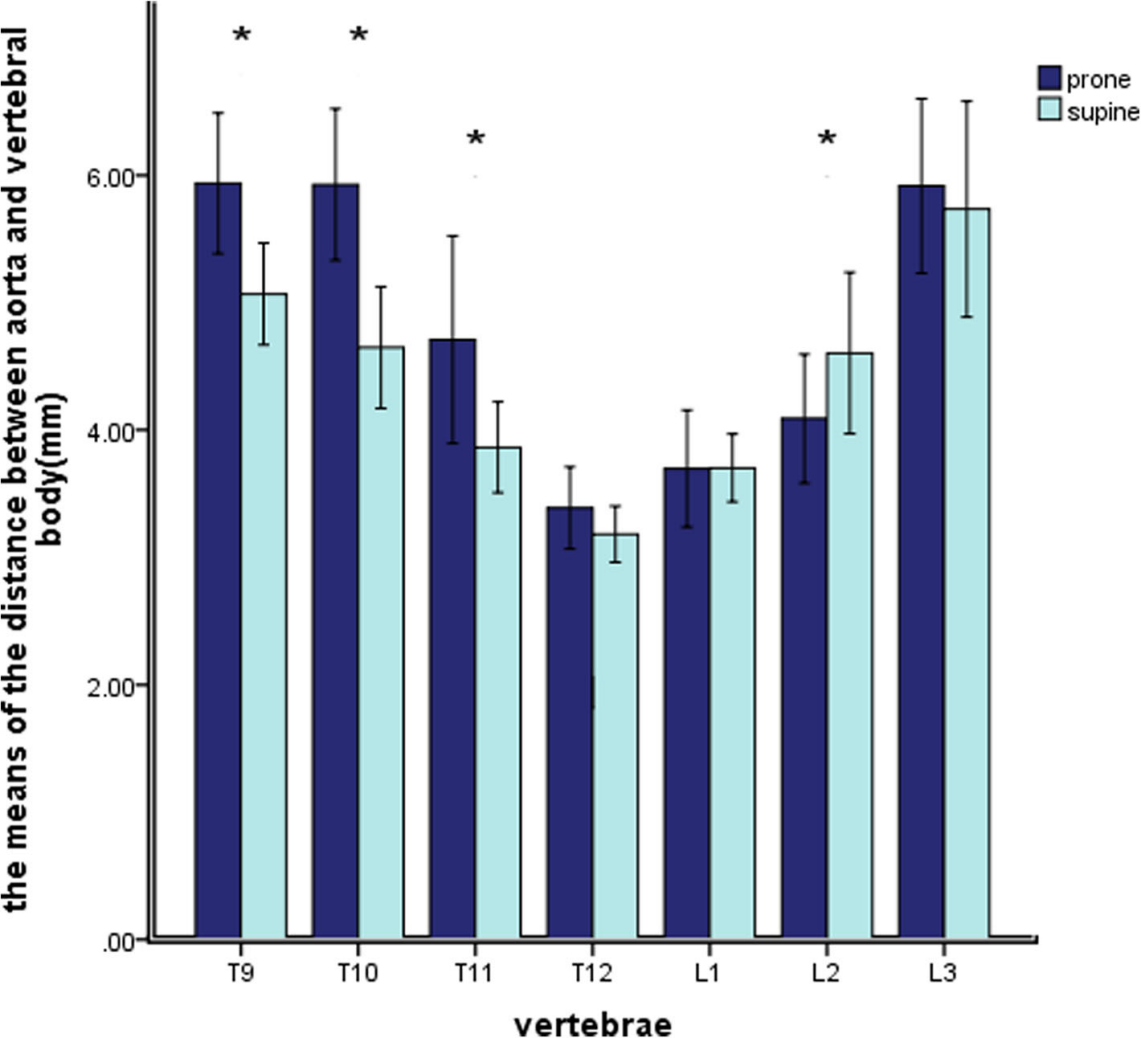
The value of AVD in the prone and the supine positions. * Means the difference in data is statistically significant

In the prone and the supine positions, the distance from the aorta to the vertebral body of the T9 ~ L3 segment decreased at first and then increased, as shown in Fig.4. In the prone position, the minimum AVD was at T12 which was 3.39 ± 0.99 mm, followed by L1 which was 3.70 ± 1.43 mm; the maximum AVD was at T9 which was 5.94 ± 1.73 mm. In the supine position, the minimum AVD was at T12 which was 3.18 ± 0.68 mm, followed by L1 which was 3.70 ± 0.83 mm; the maximum AVD was at L3 which was 5.74 ± 2.65 mm. The AVD increased more in the prone position than in the supine position, and the AVD values measured at the T9, T10, T11, L2 vertebral bodies differed significantly between the prone and the supine positions (*P* < 0.05; Table 1).

**Table 1:** The distance and relative angle between aorta and vertebral body in different positions(mm/°, Mean±SD)

level	AVD			∠AOY		
	Prone	Supine	*P* value	Prone	Supine	*P* value
T9	5.94 ± 1.73	5.07 ± 1.25	0.000*	19.47 ± 10.49	32.07 ± 11.33	0.000*
T10	5.93 ± 1.86	4.65 ± 1.50	0.000*	18.47 ± 10.93	22.79 ± 8.48	0.032*
T11	4.71 ± 2.55	3.87 ± 1.11	0.003*	11.85 ± 8.60	16.19 ± 8.40	0.000*
T12	3.39 ± 0.99	3.18 ± 0.68	0.091	14.16 ± 6.84	14.83 ± 6.98	0.412
L1	3.70 ± 1.43	3.70 ± 0.83	0.983	14.11 ± 8.71	13.80 ± 7.52	0.605
L2	4.09 ± 1.58	4.61 ± 1.98	0.001*	10.70 ± 7.61	13.58 ± 7.38	0.000*
L3	5.92 ± 2.14	5.74 ± 2.65	0.404	5.30 ± 5.68	8.85 ± 3.80	0.000*

* Means the difference in data is statistically significant

**Fig. 5:**
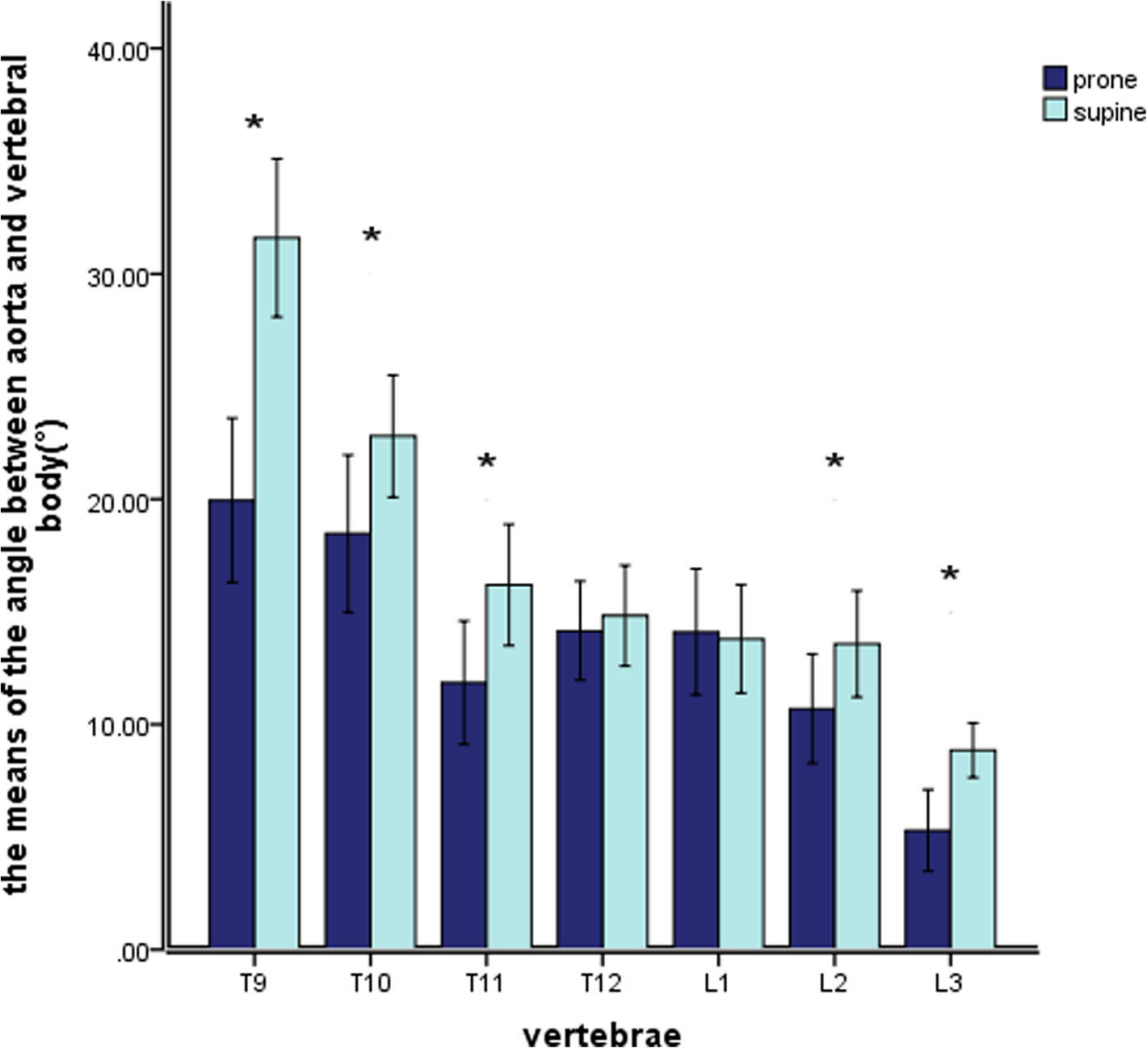
The relative Angle of the aorta and vertebral body ∠AOY in the prone position and the supine position. * Means the difference in data is statistically significant

In the prone position, the aorta of the T9-L3 segment tended to shift from the anterolateral side of the vertebral body to the anteromedial side of the vertebral body, as shown in Fig. 5, getting closer to the Y axis. At first, the ∠AOY gradually decreased from the level of T9 vertebrae (19.47 ± 10.49°) to the level of T11 vertebrae (11.85 ± 8.60°). It then increased, and a short peak appeared at the level of T12 and L1 (14.16 ± 6.84 °, 14.11 ± 8.71 °), and then decreased to the level of L3 (5.30 ± 5.68 °). In the supine position, the relative angle showed a similar result, the aorta gradually approaching the midline of the vertebral body. (Fig.5). Except for T12 and L1, there were significant differences between the angles of AOY in the prone and the supine positions (*P* < 0.05) (Table 1).

### The relative position of IVA and vertebral body (VVD /∠VOY)

**Fig. 6:**
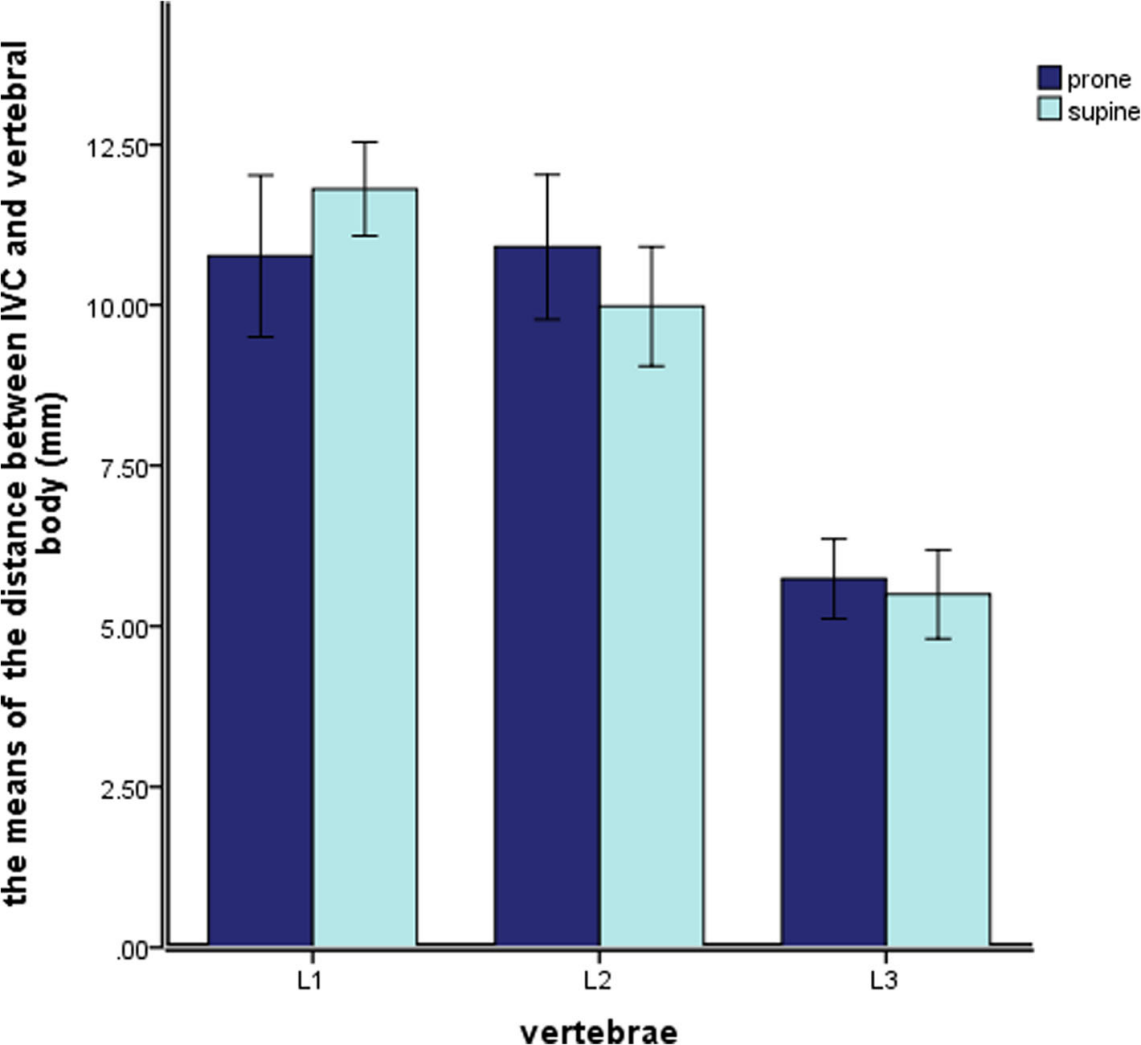
The value of VVD in the prone and the supine positions

In the prone and the supine positions, the distance between IVA and vertebral body of L1 ~ L3 segment decreased gradually (Fig.6). However, the VVD values measured at the L1–L3 vertebral bodies didn’t differ significantly between the prone and supine positions (*P* ≥ 0.05; Table 2).

**Table 2:** The shortest and relative angle between inferior vena cava and vertebral body in different positions(mm/°, Mean±SD)

level	VVD			∠VOY		
	Prone	Supine	*P* value	Prone	Supine	*P* value
L1	10.77 ± 3.93	11.81 ± 2.29	0.068	27.23 ± 4.91	26.26 ± 3.72	0.147
L2	10.91 ± 3.52	9.98 ± 2.90	0.121	29.07 ± 7.06	27.20 ± 4.93	0.054
L3	5.74 ± 1.95	5.50 ± 2.17	0.548	24.88 ± 8.21	23.21 ± 4.47	0.087

**Fig. 7:**
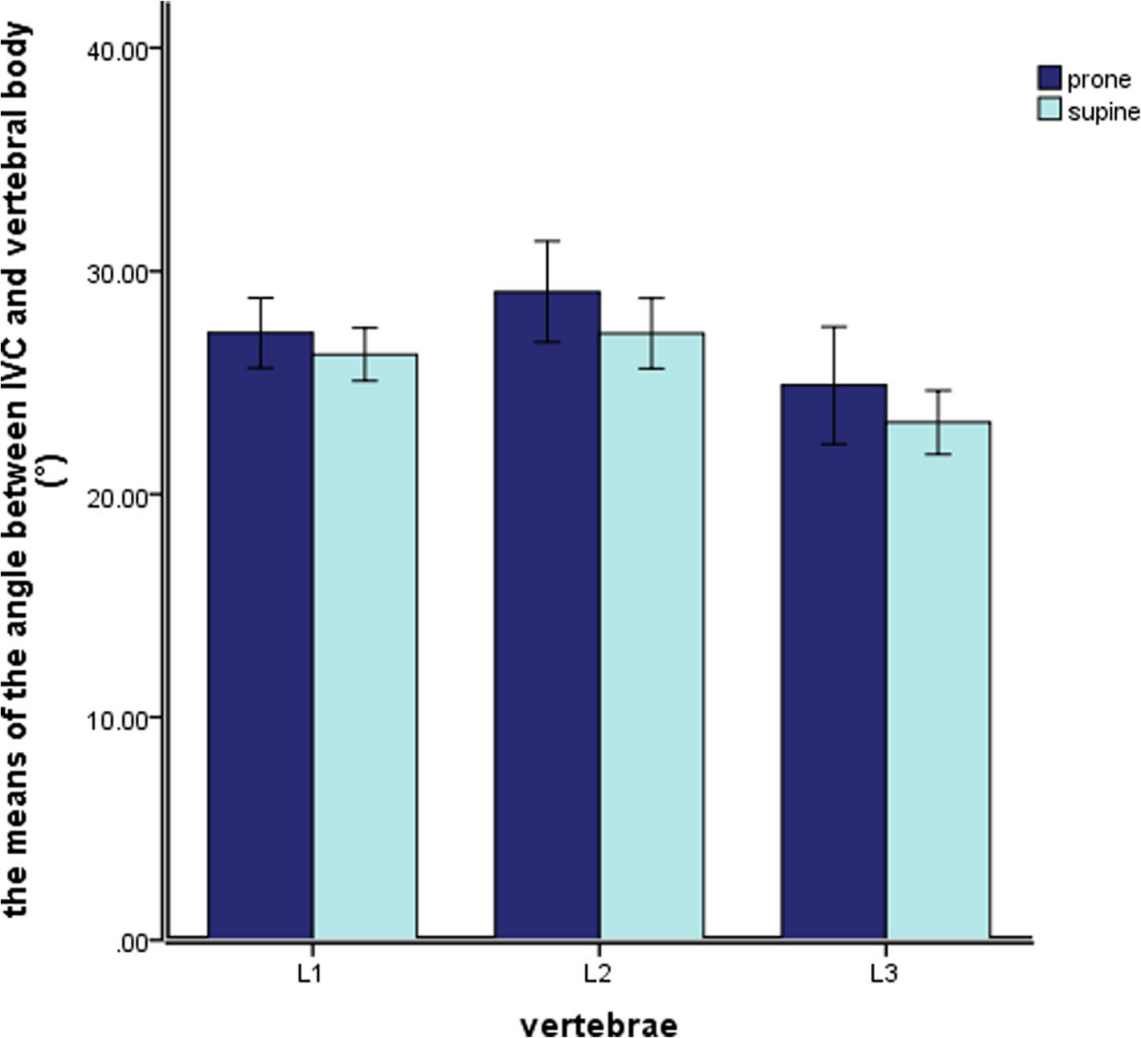
The relative angle of IVC and vertebral body ∠VOY in the prone position and the supine position

In the prone and the supine positions, the IVA of the L1-L3 segment was located in front of the right anterior side of the vertebral body (Fig. 7). However, it was limited to the 20°–30°position near the midline of the vertebral body (Y axis). There was no significant difference in the values of ∠VOY between prone position and supine position (P ≥ 0.05; Table 2).

### Intra-observer reliability

The intra-observer reliability analysis on measurements of the distance and angle showed that the correlation coefficients of AVD/VVD and∠AOY/∠VOY are 0.95 and 0.93 respectively, representing excellent reliability.

## Discussion

Bicortical fixation requires precise screw placement, as protruding screw tips can damage blood vessels [13, 14]. The experimental results show that the safe range of the protruding tips of the screw should be kept within the 3 mm. At the same time, due to the change of the relationship between blood vessels and posture, the prone position CT images can provide a more accurate and safe range of screw TSA. This is important for the safe use of pedicle screw bicortical fixation to improve the strength of fixation.

### The significance of pedicle screw bicortical insertion

It has been the focus of scholars to enhance the pullout force and internal fixation stability of pedicle screw. Clinicians wisely use bone cement augmentation, cortical bone channels, expandable pedicle and novel screw fixation. These new techniques have achieved satisfactory clinical results for the osteoporosis patient [8, 15–18]. But for patients with non-severe osteoporosis, especially those in the middle-aged and elderly with a certain amount of bone loss, surgical techniques that increase the strength of internal fixation may be a better option, particularly in increasing the diameter of the pedicle screw, the depth necessary for insertion, and the insertion angle show an advantage [19, 20].

Studies have shown that when the screw is inserted in the anterior cortex of the vertebral body but not penetrated, the fixation strength can be increased by 16%, and the anterior cortex can be broken through, which can increase the pedicle screw pullout force by 60% and fixation strength by 20–25% [21–24]. Bicortical anchorage increases the length of screw insertion and the stress is dispersed between the two cortical bones so that the fixation strength is significantly higher than that of cancellous bone [4, 5]. Its reliable fixation strength has also served in adolescent idiopathic spinal deformity or spinal deformity.

### The effect of body positions on the distance between vertebral body and the great vessel

Huitema et al. [25] proved a substantial difference in the position of the aorta relative to the spine in the prone and in the supine position (range, T4-L2), while Vaccaro et al. [26] demonstrated substantial mobility of the great vessels in different positions (range, L4-S1). We also verified the results of the aorta, and we found that the AVD of T12 is the shortest distance both in the prone and the supine positions. Considering anatomical factors, the thoracic aorta extends into the abdominal aorta from the aortic sac of the diaphragm, which is mostly located at the T12-L1 positions and is close to the vertebral body. Thus, the aorta and vertebral bodies are fixed at T12 and L1 level and will not change due to changes in body positions. Compared with the aorta, we believe that the mobility of IVC has no obvious changes in different body positions.

### Discussion on the safety distance between vertebral body and blood vessel

Sarwahi et al. [27] claimed that anterior/anterolateral protrusion is less than or equal to 4 mm on CT poses no significant risk of impingement and can be considered safe. In gross anatomy, 23 misplaced screws do not endanger any structures and the distance they protruded are less than 4 mm on CT scan [27]. Because of the large distance between the large vessels and the vertebral body in the prone position, we evaluated and measured the safe distance in the supine position with CT. The shortest distance between the aorta and the vertebra is 3.18 ± 0.68 mm at T12, and the shortest distance between the IVA and the vertebra is 5.50 ± 2.17 mm at the L3 level. Considering the large distance in the prone position and the poor visualization of soft tissue on CT images, we conservatively believe that it is safe for the protruding tip of the screw to be less than that of 3 mm. Due to individual differences, we recommend that the actual safe distance between the great vessels and the vertebral body can be measured according to our method before surgery. In addition, imaging examination shows that labial hyperosteogeny may occur on the upper and lower margin of the vertebral body in elderly patients, pushing the anterior vertebral vessels to the front of the vertebral body (Fig. 2a), increasing the safe distance between the blood vessel and vertebral body.

When evaluating misplaced screws in contact with blood vessels, the protruding tips of some of the screws are too long, which puts more degrees of impingement on the blood vessels and is more likely to cause chronic vascular injury [28]. There will also be contact between the screw and the blood vessel after the bicortical fixation. However, patients with pedicle screws in contact with major vessels may not necessarily suffer adverse sequelae [29–31]. We believe that if the tip of the screw can be controlled within the safe range, it will cause a lesser degree of screw impingement into the blood vessels and is unlikely to cause vascular damage [28].

### The effect of the angle between the vertebral body and the blood vessel on bicortical fixation of the pedicle screw

**Fig. 8:**
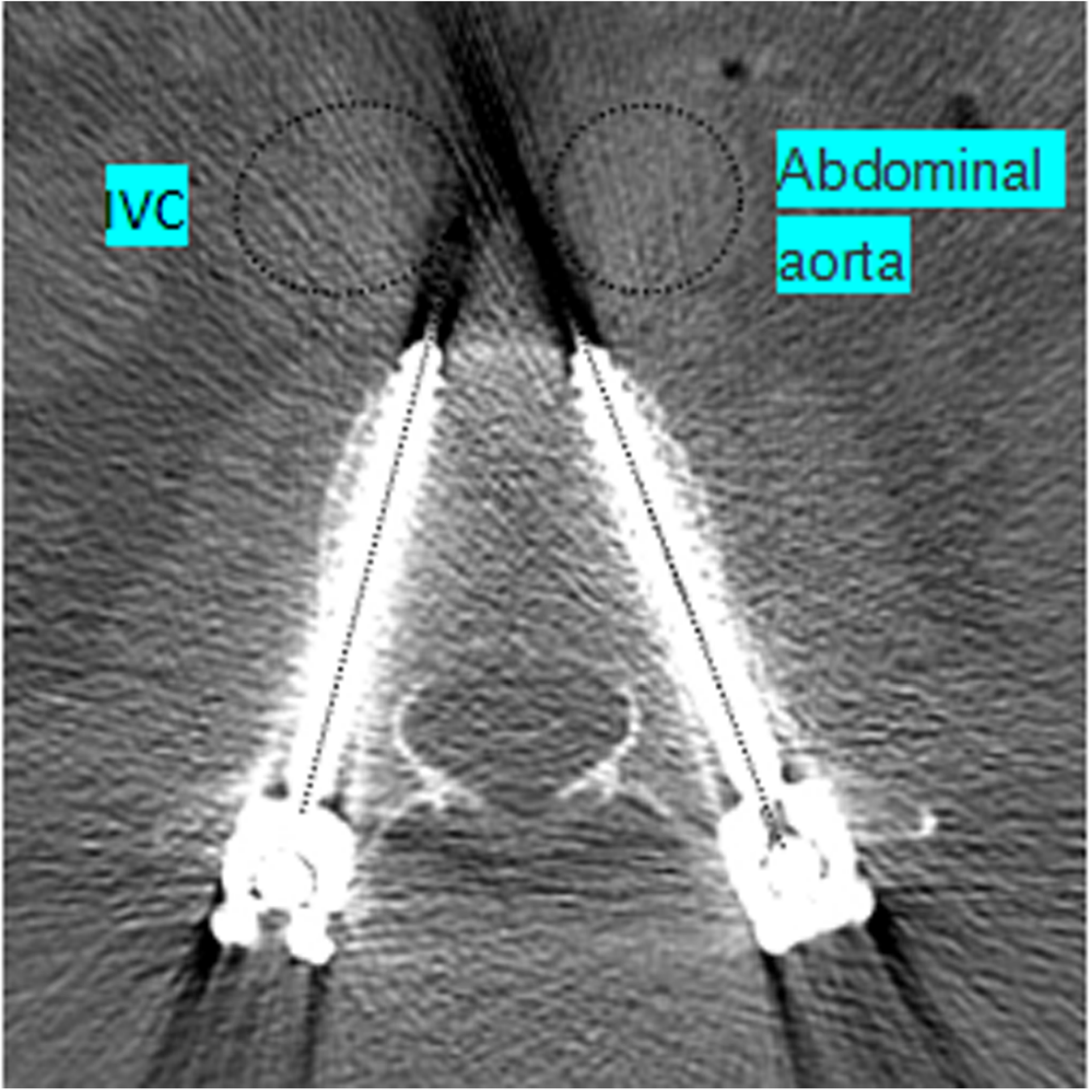
Postoperative resection of the patients with bicortical fixation revealed that the anterior vessel of the L2 vertebral body avoided the direction of the screw axis

Due to individual differences, some segmental vertebrae are close to blood vessels, and there is no obvious safe distance. However, simulating screw placement on preoperative supine and prone CT images can find the appropriate transverse screw angle (TSA), and the screw direction can avoid great vessels completely. As shown in Fig. 8, postoperative CT image of the patients with bicortical fixation presented that the great vessels of the L2 vertebral body can avoid the direction of the screw axis. Liu et.al [32] summarized the appropriate TSA of each pedicle of L1-L4. However, there is still an error in the insertion point of pedicle screw between the preoperative evaluation and the actual operation, which leads to a greater error in TSA. This requires surgeons to build their own preoperative models according to habits and to plan the correct TSA range to reduce errors.

### Limitations of this experiment

This study also had certain limitations, such as a small sample size and inevitable measurement error, although we have chosen angiography CT to minimize errors. We observed that at the level of T8-T12, the boundary between the IVC and liver tissue passing through the hepatic vena cava sulcus is not obvious. However, there is a sufficient safe distance between it and the vertebral body, which will not affect the bicortical anchorage at all.(Fig.2) In addition, we also agree that bicortical anchorage is not suitable for implantation of L4-L5 vertebrae [32], and the above relevant data were not collected in the experiment.

## Conclusion

The present data show that it is safe to ensure that the protruding tips of the screw is less than 3 mm in the treatment of thoracolumbar spinal diseases with pedicle screw bicortical anchorage. When judging the shortest distance, the measurement in the supine position is safer, but the prone position CT should be referred to when choosing the implantation direction TSA. Due to the mobility of the aorta in different postures and individual differences in anatomy, the prone position CT can help doctors to make better preoperative plans and decisions, which is of great significance for safe implementation of the bi-cortical fixation.

## Data Availability

The data used in the current study are available from the corresponding author on reasonable request.

## Abbreviations

AVD: Distance between aorta and vertebral body at the T9-L3 level
VVD: Distance between IVC and vertebral body at the L1-L3 level
AOY: Angle of position between aorta and vertebral body at the T9-L3 level
VOY: Angle of position between IVC and vertebral body at the L1-L3 level
IVC: Inferior vena cava
TSA: Transverse screw angle
